# Blastic Plasmacytoid Dendritic Cell Neoplasm: Single-Center Experience with Two Cases in One Year

**DOI:** 10.4274/tjh.2013.0404

**Published:** 2014-12-05

**Authors:** Alexandra Agapidou, Sophia Vakalopoulou, Dimitra Markala, Christina Chadjiaggelidou, Maria Tzimou, Theodosia Papadopoulou, Vasileia Garypidou

**Affiliations:** 1 Aristotle University of Thessaloniki Faculty of Medicine, Hippokratio General Hospital, Second Propaedeutic Department of Internal Medicine, Division of Hematology, Thessaloniki, Greece; 2 Theagenion Cancer Hospital, Thessaloniki, Greece

**Keywords:** Plasmacytoid dendritic cell, Leukemia, Cutaneous lesion, CD4 (+), CD56 (+)

## TO THE EDITOR

**Figure 1:**
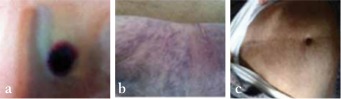
a) Skin lesion of Caucasian female patient, before treatment. b) Skin lesions of Caucasian male patient before treatment and c) after treatment (abdominal-lower genital area).

A 78-year-old Caucasian female patient presented to our department with a cutaneous lesion on her right shoulder (Figure 1a). Laboratory data disclosed mild anemia (hemoglobin: 11.3 g/dL) thrombocytopenia (139x109/L) and 30% morphologically immature atypical cells in the peripheral blood. Bone marrow aspiration showed 5% infiltration of immature blast cells with the following immunophenotype: CD45 (+), CD123 (+), CD85k (+), CD33 (-), CD14 (-), CD16 (-), CD19 (-), CD5 (-), CD10 (-), CD20 (-), CD56 (+) 20%, CD4 (+), NG2 (+). No chromosomal alterations were detected by cytogenetic analysis of the bone marrow (46,XX). Specific karyotypic aberrations were not found. She had axillary, jugular, submandibular, and supraclavicular lymphadenopathy. Cutaneous, lymph node, and bone marrow biopsy confirmed the diagnosis of blastic plasmacytoid dendritic cell neoplasm (BPDCN). She was treated with cyclophosphamide, vincristine, adriamycin, and dexamethasone (Cy-VAD) as part of an acute lymphoblastic leukemia treatment-protocol. She achieved first complete remission. Due to the highly aggressive type of leukemia, we decided to continue with induction 2 chemotherapy (etoposide-cytosine arabinoside), but she died 5 months after the first sign due to multiorgan failure.

One year later, a 75-year-old Caucasian male patient presented with a generalized purplish skin rash from the head to the lower extremities that expanded very rapidly (Figure 1b and 1c). Laboratory data revealed anemia (hemoglobin: 10.9 g/dL), thrombocytopenia (100x109/L), and 42% morphologically immature atypical cells in the peripheral blood. Bone marrow aspiration showed 88% infiltration of immature blast cells with the following immunophenotype: CD45 (+) low, CD43 (+), CD123 (+), CD56 (+), CD4 (+), CD34 (-). Computed tomography scans did not disclose pathologic lymphadenopathy. Histopathology of skin lesions showed blast cell infiltrate. Immunohistochemical analysis confirmed the presence of cells with the aforementioned immunophenotypic features. A basic immunophenotype with CD4 (+), CD56 (+), CD123 (+), and negative T, B, and NK cells led to the diagnosis of BPDCN as per the current WHO classification [1]. In the cytogenetic analysis, a pathologic karyotype was found (46,XY, del (12), (p12), del (17), (p11) [17]/46,XY [13]). He began acute myeloid leukemia-type chemotherapy with idarubicin and arabinoside-c and he achieved complete remission after induction. We changed his treatment plans and continued with CHOP due to his poor performance status, and, after 4 cycles, he still remains without clinical signs. Informed consent was obtained.

Plasmacytoid dendritic cells were first identified 50 years ago by Lennert and his associates [2]. BPDCN is a rare, highly aggressive hematopoietic malignancy that is characterized by cutaneous infiltration with or without bone marrow involvement. Its overall incidence is extremely low. The leukemic form of the disease is very rare. BPDCN predominantly affects males, and generally the elderly [3]. The majority of patients present with asymptomatic solitary or multiple cutaneous reddish-brown nodules. Clinically, this malignancy generally presents in the skin, often followed by bone marrow and blood involvement. However, any organ can be affected. The disease follows a short course and fulminant leukemia is the common terminal stage. Diagnosis is based on the expression of CD4, CD56, and CD123 in the absence of T-cell, B-cell, or myeloid markers.

Although identification of the immunophenotypic features of BPDCN has improved its recognition, this entity remains diagnostically challenging. Insufficient knowledge of this entity and inadequate immunophenotypic investigation can lead to the misdiagnosis of a different leukemia. The prognosis of patients with BPDCN is poor, with a median survival of 12 months regardless of treatment type. Acute lymphoblastic leukemia-type treatment regimens are advised and a promising initial response may occur, but this is followed by quick relapse [4]. There is also the option of bone marrow transplantation for young patients with an acceptable performance status.

In conclusion, we encountered a rare type of leukemia. The rarity of this disease does not enable prospective clinical trials to identify a better therapeutic strategy, which, at present, is based on clinicians’ experience and on cooperation among them.

**Conflict of Interest Statement**

The authors of this paper have no conflicts of interest, including specific financial interests, relationships, and/or affiliations relevant to the subject matter or materials included.
